# Lead-Free Halide Double Perovskite for High-Performance Photodetectors: Progress and Perspective

**DOI:** 10.3390/ma16124490

**Published:** 2023-06-20

**Authors:** Xiaoyan Li, Junzhe Shi, Jianjun Chen, Zuojun Tan, Hongwei Lei

**Affiliations:** College of Engineering, Huazhong Agricultural University, Wuhan 430070, China

**Keywords:** lead-free, double perovskite, photodetector

## Abstract

Lead halide perovskite has become a promising candidate for high-performance photodetectors (PDs) due to its attractive optical and electrical properties, such as high optical absorption coefficient, high carrier mobility, and long carrier diffusion length. However, the presence of highly toxic lead in these devices has limited their practical applications and even hindered their progress toward commercialization. Therefore, the scientific community has been committed to searching for low-toxic and stable perovskite-type alternative materials. Lead-free double perovskite, which is still in the preliminary stage of exploration, has achieved inspiring results in recent years. In this review, we mainly focus on two types of lead-free double perovskite based on different Pb substitution strategies, including A_2_M(I)M(III)X_6_ and A_2_M(IV)X_6_. We review the research progress and prospects of lead-free double perovskite photodetectors in the past three years. More importantly, from the perspective of optimizing the inherent defects in materials and improving device performance, we propose some feasible pathways and make an encouraging perspective for the future development of lead-free double perovskite photodetectors.

## 1. Introduction

Lead halide perovskite has the advantages of a direct bandgap, large absorption coefficient, long carrier lifetime, low defect density [1], and high solution processability, making it a good candidate for optoelectronic applications. As one of the most important optoelectronic devices, lead halide perovskite photodetectors have recently attracted enormous attention because of their potential application in optical communications, medical near-infrared imaging, military surveillance, and chemical/biological sensing. Despite the amazing achievements of lead-based photodetectors, the toxicity and chronic degradation of Pb to the human central nervous system and ecosystem cannot be underestimated [2], which has gradually become a major resistance to the commercialization of lead-based photodetectors. Therefore, it is necessary to explore intrinsically stable and environmentally friendly inorganic lead-free perovskite, which is conducive to future commercial development and is also the focus of researchers.

Among all the lead-free perovskite materials, lead-free double perovskite has emerged as a very promising candidate to address both the stability and toxicity issues in lead-based perovskites. Theoretically, lead-free double perovskite can be designed and synthesized by replacing Pb^2+^ with a low- or non-toxic metal ion. To maintain charge neutrality, two Pb^2+^ cations can usually be replaced by one monovalent metal cation M(I) and a trivalent metal cation M(III) to form the double perovskite with a general formula of A_2_M(I)M(III)X_6_, where M(I) = Na^+^, K^+^, Rb^+^, Cu^+^, Ag^+^, Au^+^, In^+^, and Tl^+^, and M(III) = Bi^3+^, Sb^3+^, In^3+^, Au^3+^, Tl^3+^, and Fe^3+^. Two Pb^2+^ ions can also be replaced by one tetravalent metal cation M(IV) to form the vacancy-ordered double perovskite with a general formula of A_2_M(IV)X_6_, where A = MA^+^, FA^+^, and Cs^+^, M(IV) = Sn^4+^, Ge^4+^, Ti^4+^, Pd^4+^, Hf^4+^, Te^4+^, Zr^4+^, and Cr^4+^, and X = Cl^−^, Br^−^, and I^−^. The A_2_M(I)M(III)X_6_ compounds are historically known as elpasolites after the mineral elpasolite, K_2_NaAlF_6_. The first reported synthesis of an elpasolite was Cs_2_Au(I)Au(III)Cl_6_ in 1922 [3]. Since the 1970s, researchers have been exploring the mixed valence state in elpasolites. In 2003, Guloy et al. successfully synthesized the first 2D perovskite A_2_[(Au(I)I_2_)(Au(III)I_4_)(I_3_)_2_] (A = 1.8 octanodiammonium or 1,7-heptadione) [4]. After extensive experiments, Hemamala et al. reported the synthesis of Cs_2_AgBiBr_6_ in 2016 [5]. Subsequently, various applications based on A_2_M(I)M(III)X_6_ perovskite have been widely developed. The origin of A_2_M(IV)X_6_ perovskite can be traced back to the synthesis of A_2_TeX_6_ (A = K^+^, Na^+^, and X = Cl^−^, Br^−^, I^−^) in 1834 [6]. Before the 1920s, researchers mainly studied its synthesis, elemental analysis, and crystal morphology. In 2014, A_2_M(IV)X_6_-type perovskite was first applied in a photovoltaic device. Regarding the development of a double perovskite-based photodetector, in 2017, Tang et al. first reported the application of ultraviolet detection and X-ray detection using the lead-free double perovskites Cs_2_AgInCl_6_ and Cs_2_AgBiBr_6_, respectively. Since then, double perovskite photodetectors have made great progress. In addition to A_2_M(IV)X_6_, vacancy-ordered double perovskites, such as Cs_2_SnI_6_, Cs_2_PdBr_6_, etc., have also been successfully implemented for the fabrication of UV photodetectors.

In this review, we discuss the properties of lead-free double perovskite materials and highlight the outstanding advances concerning lead-free double perovskite photodetectors in the last three years. Then, we emphasize the limitations of lead-free perovskite photodetector materials and devices, followed by giving a commentary on the possible solutions to solve these challenges and providing an inspiring outlook on their future directions.

## 2. Categories and Key Performance Parameters of Semiconductor-Based PDs

**Figure 1:**
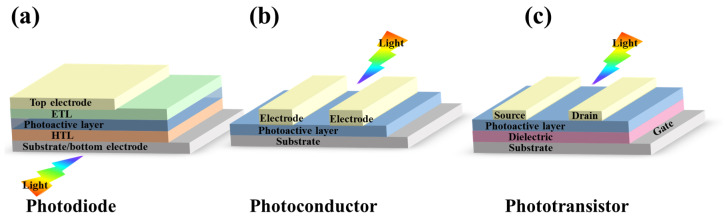
Schematic of architectures for different photodetectors. (**a**) a photodiode with a vertical structure. (**b**) a photoconductor with a lateral structure, and (**c**) a phototransistor with a bottom-gate and top-contact structure.

Semiconductor photodetectors are mainly divided into three categories according to their structures: photodiodes, photoconductors, and phototransistors. The structures of various types of detectors are shown in Figure 1. Photodiodes have narrow charge transit distance and an inner electric field; thus, they usually have fast response speed, low noise, and large detectivity, but suffer from low responsivity and external quantum efficiency (EQE). The working principle of the photodiode is as follows: when incident light with a certain energy irradiates to the junction region, electrons will transit from valence band to conduction band, resulting in the formation of a photogenerated electron-hole pair. Under the influence of the electric field, the minority carriers and the photogenerated electron holes generated in the depletion region move towards the electrodes at both ends, where they are collected by the electrodes, generating a photocurrent. In contrast, photoconductors have large responsivity/EQE because of the photoconductive gain but usually show low response time and small detectivity. The working principle of the photoconductor is similar to the photodiode except that the junction is usually a Schottky contact. Phototransistors would present balanced parameters among these three structures. For phototransistor detectors, they often exhibit a high internal photocurrent gain due to the inherent amplification function.

The key performance parameters used to characterize photodetectors are responsivity (R), detectivity (D*), response time (rise/decay time), on-off ratio, EQE, and linear dynamic range (LDR), and their definitions are expressed as follows:

1. Responsivity (R): This is a key parameter to quantify the response efficiency of photodetectors to an optical signal and is defined as the photocurrent generated by the incident light of the unit power per unit area. Its unit is A W^−1^.

$$R=\frac{I_{light}-I_{dark}}{P_{h\upsilon }S}$$

where *I_light_* is the light current generated by the light detector, *I_dark_* is the dark current, *P_hυ_* is the incident light intensity, and *S* is the effective light area.

2. Detectivity (D*): This describes the ability of detector materials to detect weak light. D* is determined by the responsivity and noise of the PD and is defined as follows:

$$D^{*}=\frac{{\left(S\Delta f\right)}^{1/2}}{I_{noise}}$$

where *S* is the effective light area of the detector, Δ*f* is the electrical bandwidth, and *I_noise_* is the total noise current of the detector. Its unit is cm Hz^1/2^W^−1^, which is equal to the unit of 1 Jones. The detection capacity can accurately reflect the photo-detection of low-intensity light, and generally speaking, the higher the D*, the better the detection performance.

3. Response time (rise/decay time): Response time reflects the response speed of the detector and is a key parameter to evaluate the performance of PDs. Generally, we can use the square wave test method to measure the optical response time of the detector. The rise time (*τ_r_*)/decay time (*τ_f_*) is defined as the rise (fall) time from 10% (90%) to 90% (10%) of the maximum current, respectively. Its unit is s.
4. On-off ratio: The on-off ratio is the ratio of the photocurrent (*I_p_*) and the dark current (*I_d_*), reflecting the photosensitivity of PDs. The higher the on-off current ratio, the higher the accuracy of the detector in detecting weak light signals.
5. EQE: This is defined as the ratio of output carriers to the number of incident photons per unit time under specific wavelength radiation, which reflects the luminous efficiency of the whole detector.

$$EQE=\frac{N_{C}}{N_{I}}=\frac{hc}{e\lambda }R$$

where *N_C_* is the number of carriers in the photocurrent, *N_I_* is the number of incident photons hitting the photodetectors, *h* is the Planck constant, *c* is the light velocity, *e* is the electronic charge, and *λ* is the wavelength of the incident light. Its unit is %.

6. LDR: This describes the region where the generated photocurrent is linearly dependent on the incident light intensity. Beyond this range, the intensity of the light signal cannot be detected and calculated precisely.

$$LDR=20\log\frac{I_{p}{}^{*}}{I_{dark}}$$

where I_p_* is the photocurrent tested under a light intensity of 1 mW cm^−2^. Its unit is dB. The lower limit of LDR is usually determined by dark current or noise. The upper limit is mainly affected by saturation effects or overload. A relatively large LDR means that the photodetector can simultaneously detect both weak and strong light signals.

## 3. Design Principle for Lead-Free Double Perovskite Materials

**Figure 2:**
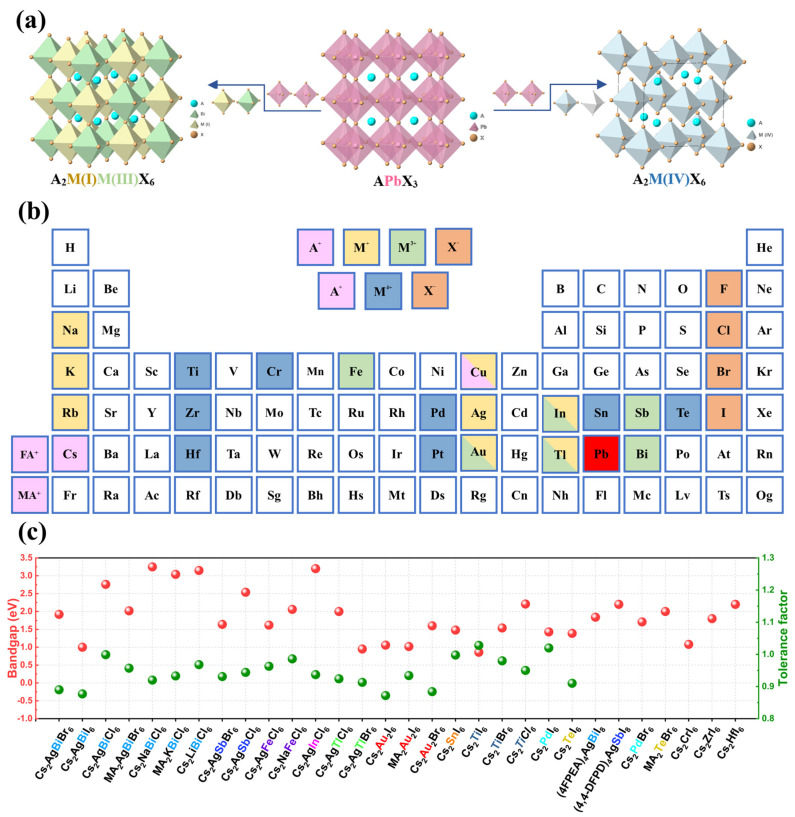
(**a**) The structure evolution from lead-based perovskite to lead-free double perovskite. (**b**) Summary of the synthesized lead-free double perovskite materials. (**c**) Summary of the bandgap and tolerance factor of the lead-free double perovskite materials mentioned in this review.

The crystal structure of lead-based halide perovskite with the general formula APbX_3_ is shown in Figure 2a, where Pb^2+^ is bonded with six X halide ions (X = I^−^, Br^−^, and Cl^−^) to form [PbX_6_]^4−^ with an octahedral structure, and each [PbX_6_]^4−^ octahedron is connected by an angle sharing mode, thus forming a 3D frame. The A-site cation, including methylammonium (MA^+^), formamidinium (FA^+^), and Cs^+^ with a large ion radius, is filled into the octahedral cavity to maintain the stability of the 3D structure. To avoid the use of toxic Pb elements, two effective strategies for replacing Pb^2+^ ions have been investigated. One strategy is to replace two Pb^2+^ ions with two heterovalent metal ions to form a double perovskite with the general formula of A_2_M(I)M(III)X_6_, whose crystal structure is shown in Figure 2a. The alternating [M(I)X_6_]^5−^ and [M(III)X_6_]^3−^ octahedra are connected by the X halogens, retaining its 3D structure. Another strategy is to replace two [PbX_6_]^4−^ with a [M(IV)X_6_]^2−^ and a [VX_6_]^6−^ octahedron where V is a vacancy, thus forming a vacancy-ordered double perovskite. Therefore, the lead-free double perovskite has a rich structural and functional diversity by combining different A, M, and X elements. It should be noted that not all metal ions can replace Pb^2+^ to form stable double perovskite. Generally, to assess the structural stability of perovskite, the tolerance factors *τ* and octahedral factors *μ* with high prediction accuracy are proposed as follows:

(1) $$t=\frac{\left(r_{A}+r_{x}\right)}{\sqrt{2}(r_{B}+r_{x})}$$

(2) $$\mu =\frac{r_{B}}{r_{x}}$$

where *r_A_*, *r_B_*, and *r_X_* represent the ionic radius of different ions and *n_A_* is the oxidation state of A. For double perovskite, the *r_B_* is the average radius of the two heterovalent metal cations. When *t* is between 0.81 and 1.11 and *μ* is between 0.44 and 0.90, we can predict that the perovskite structure is stable. In Figure 2c, we summarize the tolerance factors of various double perovskites mentioned in this review, and all of them have a suitable *t* value between 0.81 and 1.11, which indicates that they are structurally stable. In addition to the above factors, the decomposition energy is also very important and should be considered when designing stable lead-free double perovskite. Although hundreds of double perovskites have been calculated to be thermodynamically stable, a limited number of them are experimentally synthesized and investigated. We summarize the synthesized stable lead-free double perovskites in Figure 2b and their bandgaps in Figure 2c. After that, we review their applications as perovskite photodetectors in the following section.

## 4. A_2_M(I)M(III)X_6_-Based Double Perovskite Photodetectors

The quaternary double perovskites with A_2_M(I)M(III)X_6_ structures, such as Cs_2_AgBiBr_6_ [7], Cs_2_AgInCl_6_ [8], and Cs_2_AgSbCl_6_ [9], have been extensively studied recently due to their notable advantages of high inherent chemical stability, low toxicity, and long carrier lifetime. In this section, we review the basic optoelectrical performance of photodetectors by categorizing them as Bi-based, Sb-based, In-based, Fe-based, Tl-based, and Au-based double perovskite.

### 4.1. Bi-Based Double Perovskite Photodetectors

Among the A_2_M(I)M(III)X_6_ materials, Bi^3+^, which has a similar electronic configuration (6s^2^6p^0^), electronegativity, and ionic radius with Pb^2+^, is considered to be the most promising candidate to replace the Pb^2+^ ion [10]. Bi-based double perovskites, such as Cs_2_AgBiBr_6_, Cs_2_AgBiCl_6_, Cs_2_AgBiI_6_ (nanocrystals), MA_2_AgBiBr_6_, Cs_2_NaBiCl_6_, MA_2_KBiCl_6_, Cs_2_LiBiCl_6_, etc., have been widely studied in photovoltaic and photodetector fields due to their high optical absorption coefficient and long carrier recombination lifetimes. Among them, Cs_2_AgBiBr_6_ [11] stands out because of its small carrier effective mass, high humidity, and heat stability.

**Figure 3:**
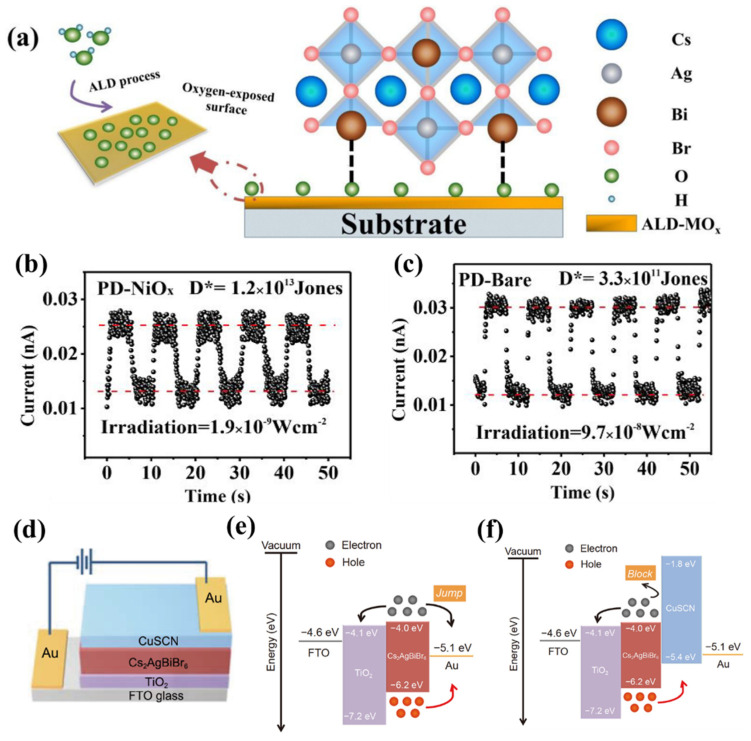
(**a**) Schematic diagram of the Bi-O interfacial interaction at the ALD-MO_x_ layer-modified substrate/Cs_2_AgBiBr_6_ interface. Reprinted with permission from Ref. [13], 2020, American Chemical Society. Typical photoresponse curves of (**b**) PD-NiO_x_ PD and (**c**) PD-Bare PD under their minimum detected irradiations. Reprinted with permission from Ref. [13], 2020, American Chemical Society. (**d**) Schematic of the Cs_2_AgBiBr_6_-based PD. Reprinted with permission from Ref. [17], 2020, Science China Press and Springer. Energy-band diagrams of the devices (**e**) with and (**f**) without CuSCN HTL. Reprinted with permission from Ref. [17], 2020, Science China Press and Springer.

In 2018, the double perovskite Cs_2_AgBiBr_6_ film prepared by the one-step spin-coating method was first used in photoconductive photodetectors, presenting a high responsivity of 7.01 A W^−1^, a specific detection rate of 5.66 × 10^11^ Jones, an on/off photocurrent ratio of 2.16 × 10^4^, and a fast response rate of 956/995 μs [12]. This work provides prospects for the development of Cs_2_AgBiBr_6_ lead-free perovskite in photoelectric detection applications. To manufacture Cs_2_AgBiBr_6_ photodetectors with a wider response range, higher responsivity, and higher detection rate, it is necessary to carefully tune its bandgap and optimize the interface between electron/hole transport layers (ETL/HTL) and the perovskite absorber layer. For example, in 2020, using H_2_O vapor and high purity N_2_ as the reactant and carrier gas, Mai et al. [13] successfully fabricated ultra-thin ALD-MO_x_ layers (ALD-TiO_2_, ALD-Al_2_O_3_, and ALD-NiO_x_) by depositing titanium tetrachloride (TiCl_4_), trimethylaluminum, and nickelene on FTO glass substrates, respectively. Then, they used them as hole transport layers to fabricate weak light photodetectors with the structure of FTO/ALD-MO_x_ interlayer/Cs_2_AgBiBr_6_/ETL/Au. Figure 3a describes the schematic diagram of Bi-O bond formation at the interface between the ALD-Al_2_O_3_ (Atomic layer deposition-Al_2_O_3_) modified substrate and Cs_2_AgBiBr_6_ film. Compared with the bare devices of photodetectors, the switching ratio was increased by 10 times, and the minimum detection radiation was reduced from 9.7 × 10^−8^ W cm^−2^ to 1.9 × 10^−9^ W cm^−2^ (Figure 3b,c), and the detection rate was improved from 3.3 × 10^11^ Jones to 1.2 × 10^13^ Jones (Figure 3b,c). This is because the Bi-O (or Ag-O) bond formed between the MO_x_ substrate and the perovskite interface contributes to the improvement of the quality of the Cs_2_AgBiBr_6_ film, resulting in films with large grain size and reduced pinholes. It is also known that proper ETL and HTL can help to optimize the perovskite morphologies and greatly improve the photocarrier transport efficiency, which is conducive to realizing high-performance photodetectors. Benefiting from the intrinsic p-type semiconductor nature and the satisfying properties of adequate energy levels, high hole mobility (1.2 × 10^−3^ cm^2^ V^−1^ s^−1^), and good thermal stability, inorganic copper thiocyanate (CuSCN) has attracted widespread attention for its application on perovskite-based devices as an HTL [14,15,16]. Inorganic thiocyanate (CuSCN) was introduced to work as the HTL for a self-powered Cs_2_AgBiBr_6_ photodetector in 2020 [17]. The device structure is shown in Figure 3d. Compared with devices without a CuSCN HTL, the light detection limit was reduced from ~7 × 10^−9^ W cm^−2^ to 1 × 10^−9^ W cm^−2^, which facilitated its usage for weak light imaging. The specific detection rate was increased from 1.74 × 10^12^ Jones to 1.03 × 10^13^ Jones and the responsivity was increased from 0.04 A W^−1^ to 0.34 A W^−1^, which was because the CuSCN hole transport layer can improve the device carrier separation and collecting efficiency. Under ideal conditions, the Cs_2_AgBiBr_6_ absorbs photo energy to generate carriers. The built-in electric field separates the carriers into electrons and holes. The electrons flow to the FTO layer, and the holes move to the Au electrode, which minimizes the recombination of carriers and generates the effective output of electrical signals. However, as illustrated in Figure 3e, since the work function of the Au electrode is located between the top of the valence band and the bottom of the conduction band of Cs_2_AgBiBr_6_, both electrons and holes will flow to the Au electrode and cause the energy losses. After introducing the CuSCN hole layer, the electron pathways to Au are blocked and the holes can be collected more efficiently, and thus the performance of the device is greatly improved.

As for the ETLs, in 2022, Shen et al. [18] introduced ZnO/SnO_2_ double ETL into Cs_2_AgBiBr_6_ double perovskite-based photodetectors. The manufacturing of the devices can be roughly divided into the following five steps: First, the ZnO seed layer was spin-coated onto the FTO substrate and annealed at 300 °C for 10 min, and the deposition process was repeated for three times. Second, the FTO substrates were soaked in zinc nitrate hydrate solution for 3 h to grow ZnO nanorods. Third, the SnO_2_ film was spin-coated and annealed at 150 °C for 30 min. Fourth, the Cs_2_AgBiBr_6_ absorption layer was deposited using a low-pressure-assisted deposition method, and finally, an Au electrode was evaporated. Compared with the detectors with a single ZnO ETL, the response rate and specific detection rate of the detector at 450 nm with the SnO_2_/ZnO double ETL were 12.7 times and 16.5 times higher, respectively. The significant improvement in device performance is attributed to the introduction of SnO_2_, which solves the mismatched energy levels problem between the ZnO ETL and perovskite film [19,20], reduces the energy loss at the interface, and optimizes electron transport and extraction. Meanwhile, the hydrophobic surface property of ZnO usually leads to a poor perovskite film [21,22]. After inserting the SnO_2_/ZnO double ETL, the interface has good wet stability, allowing for the formation of smooth and pinhole-free perovskite films [23,24]. Interestingly, ultraviolet (UV) immersion is also an effective strategy to improve photodetection performance. In 2022, Yuan and co-workers [25] performed UV immersion treatment on several Cs_2_AgBiBr_6_-based photodetectors with different structures. They use a xenon lamp as the light source for UV treatment (365 nm, 250 W). The Cs_2_AgBiBr_6_ photodetector sample was placed in front of the xenon lamp. After UV illumination, the sample was placed in the ambient for a period of time before the subsequent measurements, in order to avoid the thermal effect of UV light. The results showed that UV treatment increased the photocurrent (from ~1.0 × 10^−5^ A to ~1.5 × 10^−4^ A) and response speed (from 30.1 µs to 340 ns). To further investigate the potential mechanism responsible for this phenomenon, space-charge-limited current (SCLC) tests were performed, and the results showed that the defect density of the untreated and UV-treated devices was 8.97 × 10^16^ and 3.33 × 10^16^ cm^−3^, respectively. This finding demonstrates that continuous UV illumination can passivate the perovskite body and interface defects [26,27,28,29,30], increase the carrier concentration and/or mobility, and thus achieve the purpose of enhancing the performance of perovskite detector devices. These results drive the development of double perovskite-based photodetectors.

In addition, Cs_2_AgBiBr_6_ also has promising applications in X-ray detectors [31]. Depending on the detection principle, X-ray detectors can be divided into direct and indirect detectors. The principle of indirect X-ray detectors is to use scintillators to convert X-rays into visible light, and then use photodiodes to convert them into electrical signals and record them [32,33], which has the advantages of low cost and stable performance [34,35]. But in the photoelectric conversion process, X-ray photons need to undergo two energy conversion processes, resulting in increased energy loss. In addition, the detection sensitivity and spatial resolution are affected due to optical crosstalk during scintillation [36]. Direct-type detectors use semiconductors to convert X-ray photoelectrons directly into electronic signals, which makes the detection signal easier and faster to be collected and then the signal reproducibility is better [37]. Therefore, direct X-ray detectors have the advantages of high sensitivity, high energy resolution, and better spatial resolution for X-ray imaging. Currently, direct X-ray detectors based on perovskite are still in their infancy. According to α ∝ Z^4^/E^3^, the higher the average atomic number (Z), the higher the X-ray attenuation coefficient (α) and the higher the light absorption efficiency will be. Cs_2_AgBiBr_6_ has a large average atomic number (Cs 55, Ag 47, Bi 83), high density (4.65 g cm^−3^), and high resistivity, with superior photoelectric properties such as sensitivity and a detection limit compared to traditional materials. These characteristics make Cs_2_AgBiBr_6_ have commercial prospects in the field of X-ray detection. Currently, the X-ray detector based on Cs_2_AgBiBr_6_ has made great progress [38]. Tang’s group [39] reported an X-ray detector based on Cs_2_AgBiBr_6_ single crystals for the first time. The device achieved a low detection limit of 59.7 nGy_air_ s^−1^ by passivating the bulk phase and surface defects of Cs_2_AgBiBr_6_ single crystals by thermal annealing and surface treatment. Subsequently, Julian et al. [40] studied the electronic properties of Cs_2_AgBiBr_6_ at room temperature and liquid nitrogen temperature. They found that lowering the temperature from room temperature to liquid nitrogen temperature can increase the carrier lifetime and resistivity of Cs_2_AgBiBr_6_ perovskite, which led to an increase in detector sensitivity from 316 μC Gy_air_^−1^ cm^−2^ to 988 μC Gy_air_^−1^ cm^−2^. Although low-temperature technology requires high experimental conditions, this result provides ideas on how to improve the detector sensitivity. In addition, Shao et al. [41] synthesized Cs_2_AgBiBr_5.933_Cl_0.067_ SC by doping Cl ions in Cs_2_AgBiBr_6_, which exhibited low trap density of states and high carrier mobility. It achieved excellent device performance in X-ray detection, including a superior X-ray detection sensitivity of 714 μC Gy_air_^−1^ cm^−2^, and a minimum dose rate as low as 36.48 nGy_air_ s^−1^. Currently, achieving long-term operational stability is the future research direction of Cs_2_AgBiBr_6_-based double perovskite X-ray detectors [42]. Cs_2_AgBiBr_6_ is prone to defect formation, such as anti-situ defects (AgBi or BiAg) and vacancy defects (Bi or Br vacancies) [43], which severely limits the preparation of X-ray detectors with lower detection limits and higher sensitivity, as well as the operational reliability of devices. Considering these parameters, further research on X-ray detectors based on Cs_2_AgBiBr_6_ is needed.

### 4.2. Other (Sb^3+^, Fe^3+^, In^3+^, Tl^3+^, Au^3+^-Based) Double Perovskite Photodetectors

Apart from the Bi^3+^ ion, the Sb^3+^ (5s^2^5p^0^) ion also has similar electronic structures to the Pb^2+^ ion. Theoretically, it can be used as a candidate to replace the Pb^2+^ ion. At present, Cs_2_AgSbBr_6_ [44] (bandgap of 1.64 eV) and Cs_2_AgSbCl_6_ [45] (bandgap of 2.54 eV) have been successfully synthesized. Currently, there is no report on Cs_2_AgSbX_6_-based photodetectors and this is because of the high formation energy of Cs_2_AgSbX_6_ and the small ionic radius of Sb^3+^. These crystals are accompanied by large amounts of secondary phases such as Cs_3_Sb_2_Br_9_ and Cs_2_AgBr_3_ and unreacted AgBr [46]. Moreover, Wang et al. [47] used 4,4 difluoropiperidinium chloride, Ag_2_O, and Sb_2_O_3_ as raw materials and HI as solvent, and the precursor solution was heated and stirred at 373 K until a clear solution was formed. Then, the solution was cooled down to room temperature at a rate of 2 K/day to prepare (4,4-DFPD)_4_AgSbI_8_ (4,4-DFPD = 4,4-difluoropyridine) 2D perovskite single crystals which were successfully used in X-ray detection. The detector possesses promising X-ray responsivity with a sensitivity as high as 704.8 µC Gy_air_^−1^ cm^−2^ at 100 V bias and a detection limit as low as 0.36 µGy_air_ s^−1^ at 10 V bias [47]. In recent years, Fe-based double perovskites such as Cs_2_AgFeCl_6_ and Cs_2_NaFeCl_6_ have attracted increasing attention with their excellent optical absorption property [48].

Unfortunately, even at low temperatures, there is no detectable PL in the Cs_2_AgFeCl_6_ or Cs_2_NaFeCl_6_ crystal which would limit its application in optoelectronic fields. As a member of the A_2_M(I)M(III)X_6_ double perovskite family, Cs_2_AgInCl_6_ has a large direct bandgap (3.2 eV), which determines that Cs_2_AgInCl_6_ mainly absorbs light with a wavelength less than 400 nm, so it can be used to manufacture UV photodetectors [49]. Tang and co-workers designed and manufactured a photoconductive planar UV detector based on Cs_2_AgInCl_6_ single crystals [50], which showed a current switching ratio of ~500, a light response speed of ~1 ms, a low dark current (~10 pA at 5 V bias), and a high detection rate (~10^12^ Jones). The Cs_2_AgInCl_6_ single crystal shows two absorption edges at 384 and 595 nm, respectively, corresponding to inter-band transition (from CBM to VBM-2) and parity-induced forbidden transition (from CBM to VBM). The parity-forbidden transition in the direct bandgap system leads to very weak emission of Cs_2_AgInCl_6_, which hinders its practical application in optoelectronics. Alloying or doping in Cs_2_AgInCl_6_ can break the parity-forbidden transition, change the bandgap, improve the luminous efficiency, and ultimately improve the detection performance. At present, the doping of transition metal ions such as Cu^2+^ [51], Mn^2+^ [52], and lanthanide elements has been explored. In 2022, Qiu and co-workers [53] synthesized Cs_2_Na_x_Ag_1-x_InCl_6_ (X = 0.16, 0.4, 0.58, 0.78, 1) nanocrystals with different amounts of sodium doping by hydrothermal method. The introduction of Na^+^ improved the photoluminescence intensity of Cs_2_AgInCl_6_, where x = 0.78 has the highest luminous intensity. The broadband emission range of the sample is 400~800 nm, covering the whole visible spectrum. The transparent composite film prepared based on Cs_2_Na_0.78_Ag_0.22_InCl_6_ crystals mixed with PMMA can capture weak solar blind near-ultraviolet light, convert it to visible light, and display the captured signal on a digital oscilloscope after being sensed by a light correlation resistor. The device has a response speed of about 0.5 s and has high detection stability. Tl^3+^, which is in the same main group as In^3+^, can also be used as the M-site ion of A_2_M(I)M(III)X_6_ double perovskite. In 2018, Karunadasa and co-workers [54] first synthesized double perovskite Cs_2_AgTlCl_6_ and Cs_2_AgTlBr_6_ by the slow cooling crystallization method. Both of them have a cubic double perovskite structure with *Fm* $\bar{3}$ *m* symmetry at room temperature. Cs_2_AgTlBr_6_ possesses the smallest reported direct bandgap for a halide perovskite at 0.95 eV. Meanwhile, Cs_2_AgTlBr_6_ and Cs_2_AgTlCl_6_ have good environmental stability, with no change in their XRD peak position when exposed to light (0.75 suns, 60 °C) and humid air (55% relative humidity) for 40 days. Preliminary results show that Cs_2_AgTlX_6_ has the potential to convert light into electricity [55]. However, Tl is highly toxic and this would hinder its future development. In addition to the above double perovskite, gold-based double perovskite has attracted researchers’ attention due to its narrow direct bandgap (1.06 eV) [56]. In the gold-based double perovskite structure, gold and halogen ions form a compressed octahedron [Au^+^X_6_]^5−^ and elongated [Au^3+^X_6_]^3−^ octahedron, respectively. The two octahedrons form a tetragonal Cs_2_Au_2_X_6_ through the shared connection of halogen ions. The electronic band structure shows that Cs_2_Au_2_X_6_ has a slight indirect bandgap, and the difference between the indirect bandgap and the optically permissible direct bandgap is very small, only about 0.03 eV [57]. Like Fe-based double perovskite, Cs_2_Au_2_I_6_ also has no detectable PL, and the cause of this phenomenon remains to be investigated. Preliminary results have demonstrated that gold-based double perovskite can convert light into electricity and deliver a solar-to-electricity efficiency for MA_2_Au_2_I_6_ [58].

## 5. A_2_M(IV)X_6_-Based Double Perovskite Photodetectors

Since Sn^4+^ single crystals were first reported in 2013, an increasing number of novel A_2_M(IV)X_6_ double perovskites with exciting optoelectronic properties have emerged [59]. There are high hopes for the great potential of vacancy-ordered A_2_M(IV)X_6_ in various photoelectric applications. In this section, we discuss the fundamental properties of double perovskites with A_2_M(IV)X_6_ structures and their recent progress in the field of photodetectors.

### 5.1. Sn-Based Double Perovskite Photodetectors

Among all bivalent substitution elements for Pb^2+^, Sn^2+^ is considered one of the most promising candidates due to its similar electronic structure and ionic radius (1.35 Å for Sn^2+^ and 1.49 Å for Pb^2+^) with Pb^2+^. However, the 5s orbitals in the Sn^2+^ cations are susceptible to oxidation and will generate a large density of oxygen vacancies, thus limiting the charge transport and even leading to material degradation [60]. Therefore, Sn^2+^-based perovskite is extremely unstable in the ambient atmosphere. In contrast, the Sn^4+^ ion exhibits excellent stability to moisture and light due to the filled 4d^10^ electron orbitals and high electronegativity. Recently, vacancy-ordered double perovskites A_2_SnX_6_, which have direct bandgaps, adjustable light response ranges, and high air and moisture stability, have attracted increasing attention in optoelectronic detection applications.

**Figure 4:**
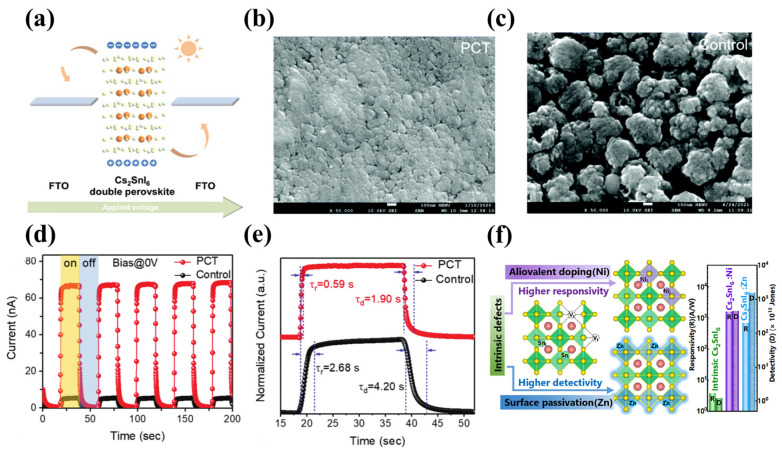
Cs_2_SnI_6_-based photodetectors. (**a**) Schematic working mechanism. Reprinted with permission from Ref. [61], 2019, Wiley. SEM images of the PCT-Cs_2_SnI_6_ film (**b**) and control sample (**c**). Reprinted with permission from Ref. [64], 2021, Royal Society of Chemistry. (**d**) Photo-switching characteristics of the PCT-Cs_2_SnI_6_ PD and control device at zero bias with periodic illumination. Reprinted with permission from Ref. [64], 2021, Royal Society of Chemistry. (**e**) Rising and falling edges of one response cycle for determining the response time of the Cs_2_SnI_6_ PDs. Reprinted with permission from Ref. [64], 2021, Royal Society of Chemistry. (**f**) A schematic diagram of the crystal structure of Cs_2_SnI_6_ perovskites with Zn ion and Ni ion incorporation. Reprinted with permission from Ref. [65], 2023, American Chemical Society.

Han et al. [61] synthesized lead-free double perovskite Cs_2_SnX_6_ (X = I and Br) with high crystallinity and high yield by a hydrothermal method in 2019 and fabricated a double-ended photodetector with a simple structure of FTO/Cs_2_SnI_6_/FTO to explore the potential applications of Cs_2_SnI_6_ for photodetection. As shown in Figure 4a, the Cs_2_SnI_6_ film will generate free carriers under appropriate light irradiation, and then the carriers are collected by the opposite electrodes with the help of the external electric field, thus producing an efficient photoresponse current. The current–time (I-T) response of the Cs_2_SnI_6_ photodetector was tested under intermittent irradiation and 3V bias voltage. The results showed that the rise time (t_r_) and decay time (t_d_) of the Cs_2_SnI_6_ photodetector were less than 100 ms. More importantly, the unencapsulated Cs_2_SnI_6_ photodetector showed superior stability to moisture and light because of the stable crystal structure and chemical composition of Cs_2_SnI_6_.

To realize high-performance photodetectors, it is vital to fabricate high-quality pure-phase Cs_2_SnI_6_. So far, the main methods for preparing the Cs_2_SnI_6_ thin films are vapor deposition and solution deposition [62]. The evaporation method has strict requirements for reaction conditions and precursors, which usually include high temperature, high vacuum, and long reaction time. In addition, the SnI_4_ will be thermally decomposed during thermal evaporation. In contrast, the one-step spin-coating is an efficient and low-cost method for preparing Cs_2_SnI_6_ films. However, the solution-deposited films usually present imperfect morphologies with pinholes, impurities, and low film coverage which is believed to be caused by the too-slow nucleation and crystallization. Therefore, optimizing the reaction conditions and preparing high-quality Cs_2_SnI_6_ films or crystals are the first task to achieve high-performance photodetectors. Krishnaiah et al. [63] optimized the preparation process of Cs_2_SnI_6_ thin film. It was found that at a specific environmental condition, i.e., the relative humidity was less than 40% and the annealing temperature was kept at 75 °C, the Cs_2_SnI_6_ perovskite thin film became pure and dense. In all other conditions with higher RH% (50%, 60%, 80%) and annealing temperature, the impurity CsI phase was detected together with the Cs_2_SnI_6_ phase for the fabricated films. At 1 V bias voltage, the maximum R_ph_ and D_ph_ for Cs_2_SnI_6_ PD were 6 mA W^−1^ and 2.00 × 10^9^ Jones, respectively. To further improve the performance of the Cs_2_SnI_6_ detector, Tan and co-workers [64] innovatively developed a precursor compensation treatment (PCT) technique by spin-coating SnI_4_/isopropanol solution on the as-prepared Cs_2_SnI_6_ film, which provided an I-rich environment for the growth of the Cs_2_SnI_6_ film, compensating for the SnI_4_ loss and suppressing the formation of inherent defects. As shown in Figure 4b,c, the Cs_2_SnI_6_-PTC films were dense and smooth with fewer defects and higher coverage compared to the unoptimized films. Photodetectors with a configuration of FTO/c-TiO_2_/Cs_2_SnI_6_/spiro OMeTAD/Au were fabricated. Under zero bias voltage and light illumination with different intensities, the maximum response of PCT-Cs_2_SnI_6_ PD reached 1.07 mA W^−1^, and the specific detection rate was 6.03 × 10^10^ Jones. Both of these were higher than the control group (R = 0.0059 mA W^−1^, D* = 2.94 × 10^10^ Jones), suggesting that the ability to detect white light and weak light signals was enhanced. Moreover, compared with the control group, the PCT-Cs_2_SnI_6_ PD exhibited a higher switching ratio (Figure 4d) and faster response speed (Figure 4e). It was noted that surface defects in double perovskite were a cause of its reduced resistance and increased dark current, which also affected the performance of detector devices. Very recently, Shen and his colleagues [65] reported that introducing metal ions of Zn^2+^ and Ni^2+^ into Cs_2_SnI_6_ can reduce the surface defects of perovskite. The Cs_2_SnI_6_ nanochip detector based on metal ion doping showed a spectral response at 400–900 nm. Specifically, the Cs_2_SnI_6_ device doped with Ni^2+^ had an excellent response rate of 1.6 × 10^3^ A W^−1^, and the Cs_2_SnI_6_ device doped with Zn^2+^ had a high detection rate of 1.56 × 10^13^ Jones (Figure 4f). The performance of the Cs_2_SnI_6_ device based on metal ion doping exceeded that of other lead-free perovskite-based photodetectors, and even reached the level of the best reported lead-based perovskite photodetectors. This is inspiring for the research and development of lead-free double perovskite-based photodetectors.

### 5.2. Other (Ti^4+^, Pd^4+^, Hf^4+^, Te^4+^, Cr^4+^, Zr^4+^-Based) Double Perovskite Photodetectors

Since many transition metals have stable +4 oxidation states, and meanwhile are non-toxic or have low toxicity, researchers have explored the possibility of replacing the Sn^4+^ in Cs_2_SnI_6_ with appropriate transition metal cations. Among them, Ti^4+^, Pd^4+^, Hf^4+^, Te^4+^, Cr^4+^, and Zr^4+^ have been proved to be thermodynamically stable and have been synthesized.

**Figure 5:**
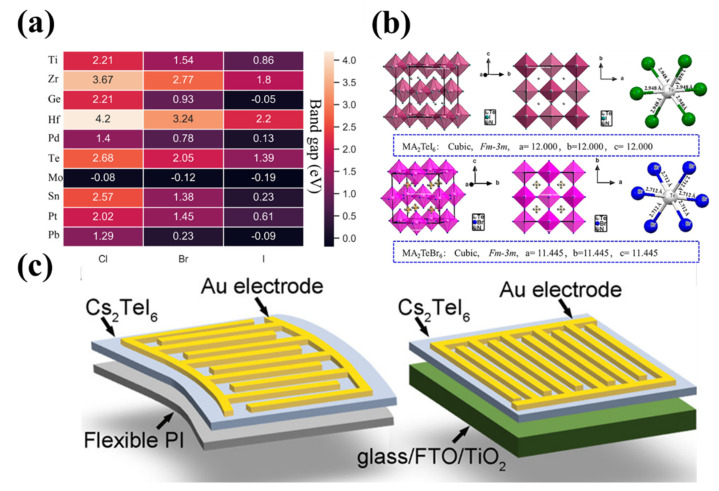
(**a**) Results of bandgap prescreening of the 30 studied perovskites. Reprinted with permission from Ref. [66], 2022, Wiley. (**b**) The crystal structures of MA_2_TeI_6_ and MA_2_TeBr_6_. Reprinted with permission from Ref. [69], 2019, American Chemical Society. (**c**) Sketches of Cs_2_TeI_6_ detector structures based on the flexible PI and rigid FTO substrates. Reprinted with permission from Ref. [71], 2021, American Chemical Society.

In 2022, Ye and colleagues [66] screened 10 covalent substituents of M-site metal ions in vacancy-ordered double perovskite Cs_2_M(IV)X_6_ using the density functional theory (DFT). Among them, for Ti, Zr, and Hf elements in the same group, the bandgap (0.86 eV, 1.8 eV, 2.2 eV (X = I)) increases with the increase of the M-site cation radius. The transition from chloride to bromide and then to iodide gradually narrows the bandgap, as shown in Figure 5a. Ti-based double perovskite is non-toxic, earth-abundant, and biocompatible. The DFT calculation shows that the bandgap of the Cs_2_TiX_6_ material is between 1.5 and 2.96 eV, and has a large absorption coefficient in the visible light range. In detail, the maximum absorption coefficient of Cs_2_TiBr_6_ at 422 nm is about 3 × 10^5^ cm^−1^, the absorption coefficient of Cs_2_TiI_6_ at 526 nm is 2.35 × 10^5^ cm^−1^, and the absorption coefficient of Cs_2_TiCl_6_ at 380 nm is 2.22 × 10^5^ cm^−1^. In addition, Ti-based double perovskite has high optical conductivity (10^3^ Ω^−1^ cm^−1^) and a good refractive index in the visible range. These optical properties make it promising for optoelectronic applications [67]. For instance, the Cs_2_TiBr_6_-based solar cell has been demonstrated with a photo-to-electric conversion efficiency of 3.3% [68]. Its corresponding photo-detecting application is still waiting to be verified. The A_2_TeX_6_ compound (A = MA, FA, or BA; X = Br^−^ or I^−^; MA = CH_3_NH_3_; FA = CH(NH_2_)_2_; BA = benzylamine), as a member of the vacancy-ordered double perovskite, has also attracted the attention of researchers. It has an adjustable bandgap (1.42–2.02 eV), high mobility (~65 cm^2^ V^−1^ s^−1^), long carrier diffusion length (38 μm), and good thermal stability. [69] The single-crystal XRD patterns indicate that the crystal structure of both MA_2_TeI_6_ and MA_2_TeBr_6_ belongs to the cubic *Fm* $\bar{3}$ *m* space group. [70] Their crystal structures are shown in Figure 5b [69]. Guo and co-workers [71] prepared Cs_2_TeI_6_ films on flexible polyimide (PI) substrates by electrospray and used them in X-ray detectors. The device structures are shown in Figure 5c, which are PI/Cs_2_TeI_6_/Au and glass/FTO/TiO_2_/Cs_2_TeI_6_/Au, respectively. The flexible and rigid devices showed a high sensitivity of 59.28 and 76.27 μC Gy_air_^−1^ cm^−2^ at 5 V bias voltage and 20 kV X-ray, respectively. In addition, Rb_2_TeI_6_ prepared by a dry process has been successfully applied in photodetectors. Under 450nm light, the photodetector based on Rb_2_TeI_6_ showed a light response of 1.4 mA W^−1^, a light detection performance of approximately 10^10^ Jones, and a response time of 16.4 ms/19.2 ms [72]. Cs_2_CrI_6_, as a new vacancy-ordered perovskite, possesses more excellent stability and optical absorption, suitable bandgap (1.08 eV), higher mobility (~10^3^ cm^2^/V), and lower capture cross-section compared to the MAPbI_3_ [73]. Based on the first-principle calculations, the calculated electron and hole mobilities of Cs_2_CrI_6_ (μ_n_ = 7.24 × 10^3^ cm^2^/V, μ_p_ = 6.57 × 10^3^ cm^2^/V) are larger than those of MAPbI_3_ (μ_n_ = 24.8 cm^2^/V, μ_p_ = 293 cm^2^/V) [74,75]. The superior mobility suggests that Cs_2_CrI_6_ may possess greater potential than MAPbI_3_ in the application of photodetectors. We note that many novel lead-free double perovskite materials emerge and have been utilized in photoelectric applications. We therefore summarize the device structures and the corresponding performances of the photodetectors involved in this paper in Table 1 for a better comparison.

## 6. Challenges and Perspective for Lead-Free Double Perovskite-Based PDs

**Figure 6:**
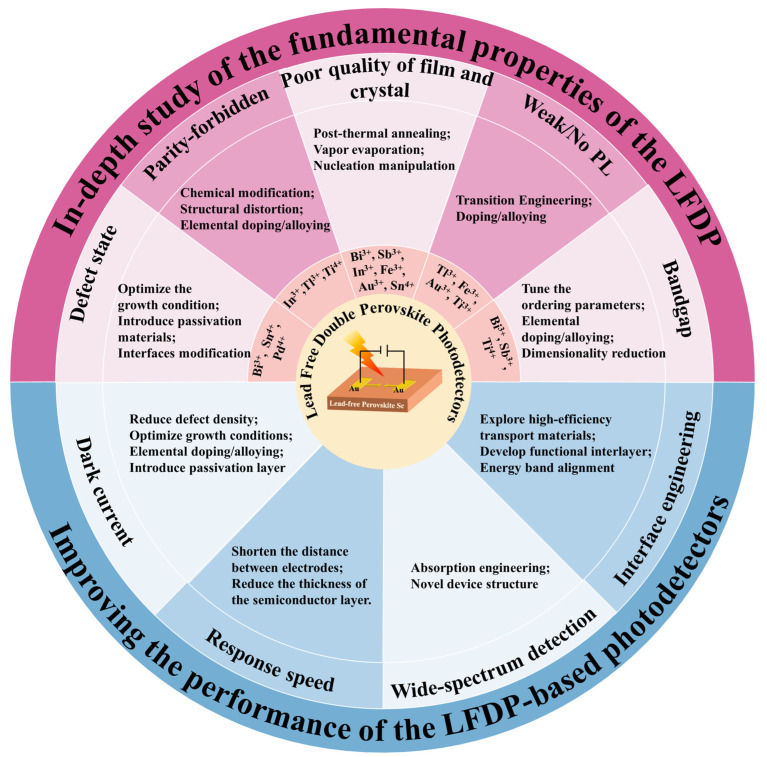
Summary of the challenges and viable strategies of the lead-free double perovskite photodetectors.

As mentioned in the previous section, lead-free double perovskite photodetectors have received a lot of research attention in recent years and have achieved impressive progress. Despite the tremendous progress in photodetection, there are still many challenges in the future direction of this field, which may hinder the development of lead-free double perovskite and limit its potential applications. We systematically summarize the challenges faced by the use of various types of lead-free double perovskite to fabricate higher-performance photodetectors, and we propose the corresponding solutions, as summarized in Figure 6 and discussed as follows:

(1) In-depth understanding of the fundamental material properties of the lead-free double perovskite is needed. Compared with the well-studied lead-based perovskite, lead-free double perovskite, as a new branch of perovskite materials, is undoubtedly underinvested and many aspects have not been effectively or deeply investigated. To explore the potential of lead-free double perovskite, researchers need to investigate more deeply its potential mechanism, crystal structure, and optoelectronic properties.

We summarize five material challenges that limit the wide application of double perovskite in optoelectronics. First, compared to the conventional lead-based perovskite, double perovskite has more deep-level defect states in the lattice and bulk phases, which reduces its carrier mobility and lifetime. To understand the mechanism of defect traps in the structure, the basic charge carrier dynamics need to be studied in detail. The growth condition needs to be carefully tuned to obtain high-quality perovskite absorbers. Meanwhile, the methods of the defect passivation and surface modification of lead-free perovskite should be explored. For example, the incorporation of non-volatile Lewis-base molecules such as urea and thiourea into the perovskite precursor solution can regulate crystal growth and cause single crystals to precipitate along grain boundaries to passivate defects [86].

Second, for some lead-free double perovskites with indirect bandgaps, such as Bi-based, Sb-based, and Ti-based, they require phonon emission or absorption to maintain momentum, which lead to their relatively low absorption coefficients [87]. Bandgap engineering can modulate the optoelectronic properties of lead-free double perovskite to make the transition from indirect bandgap to direct bandgap. The engineering strategy mainly consists of elemental doping/alloying and tuning the ordering parameters. Doping and alloying are the most powerful methods for adjusting the optical, electrical, and structural properties of perovskite [88]. For example, through alloying with In^3+^ or Sb^3+^, the indirect bandgap of bulk Cs_2_AgBiBr_6_ (2.12 eV) has been altered to 2.27 and 1.86 eV, respectively, which is caused by the different atomic configurations of In and Sb. However, most of the In and Sb alloyed samples show reduced PL intensity, suggesting the presence of relatively deep defect states. The ordered-disorder parameters can be intentionally adjusted through growth regulation or external pressure treatment, resulting in changes in the bandgap [88]. Direct bandgaps can be also achieved from some low-dimension structures such as the (BA)_4_AgBiBr_8_ [89] and (AE2T)_2_AgBiI_8_ [90]. In addition, doping VA group elements (Sb^3+^, Bi^3+^) in the Cs_2_NaInCl_6_ material can significantly improve the light absorption intensity of the material in the near-ultraviolet region, which can be applied to wide-spectrum photodetection. [91]

Third, for double perovskite compounds with direct but parity-forbidden transition gaps, such as Cs_2_AgInCl_6_, Cs_2_AgTlCl_6_, Cs_2_NaInCl_6_, and Cs_2_TiBr_6_, they have poor absorption ability for photons with energies close to the bandgap. Chemical modification, structural distortion or elemental doping can be used to eliminate or break the optical transition selection rules and make them more suitable for photoelectrical applications. For example, alloying Na^+^ into Cs_2_AgInCl_6_ can efficiently break the parity-forbidden transition of the host material [92].

Fourth, the preparation of dense and uniform high-quality thin films is a major challenge for almost all lead-free double perovskite detectors. For Bi^3+^-based, Sn^4+^-based, In^3+^-based, Au^3+^-based, and Sb^3+^-based double perovskite, their precursor materials are mostly insoluble in organic solvents, thus making it difficult to obtain pure-phase films when preparing films by the spin-coating method. We should explore more effective synthesis strategies to prepare high-quality lead-free double perovskite with optimized morphologies. Among them, proper post-thermal annealing treatment of spin-coated films is an effective option. In addition, using vacuum thermal evaporation to prepare double perovskite thin films is also feasible. For example, the self-powered UV photodetector, which is based on Cs_2_AgBiBr_6_ thin film prepared by sequential vacuum evaporation method, has obtained a high on/off ratio of 6.6 × 10^3^ and a fast response time of 6.13/28.02 μs [85]. In addition, the synthesis of pure-phase and large-size single crystals is also crucial for the development of lead-free double perovskite detectors. Compared with thin films, bulk single crystals display the advantages of fewer defects and high stability. For Sb^3+^-based double perovskite with a high formation energy, the synthesis of pure-phase single crystals remains to be explored. Accordingly, we should deepen our understanding of the crystallization mechanism involved in the synthesis process, which can help to improve the morphology of the films and single crystals, thus optimizing the device performance.

Finally, for some double perovskites which have very weak or no PL emission, such as Fe^3+^-based, Au^3+^-based, and Ti^4+^-based [93] double perovskites, we need to understand the possible reasons behind these properties such as the indirect bandgap, and the parity-forbidden direct/indirect transition, and then solve the problems using strategies such as doping, alloying, and tailoring the dimensionality. In the meanwhile, it is also essential to invest more efforts into the exploration of new materials with other structures. For example, the lead-free stable oxide double perovskite A_2_M(III)M(V)O_6_ (A = Ca, Sr, Ba; M(V) = V^5+^, Nb^5+^, or Ta^5+^), which is transformed from prototype perovskite oxide CaTiO_3_, has been used as an absorbing layer material for photodetectors. Among them, a self-powered ITO/BBNO/Ag photodetector based on Ba_2_Bi_1.5_Nb_0.5_O_6_ (Eg = 1.37 eV) has shown an optical response in the range of 365–760 nm and exhibited a photocurrent of 59.2 mA cm^−2^ and a response of 78.8 mA W^−1^ [94]. Another structurally similar calcium niobate Ca_2_Nb_2.5_Ta_0.5_O_10_ nanosheet is also used as the photoactive layer of the detector, and the device shows a switching ratio of 5.6 × 10^4^ and a responsiveness of up to 469.5 A W^−1^ at 1 V bias and 295 nm illumination [95]. In addition to the above oxide perovskite, the A_3_M(I)M(III)X_7_ 2D materials also deserve more in-depth study. Very recently, researchers have conducted a detailed study of Cs_3_AgBiBr_7_ based on the first principles. The results show that the material exhibits significant light absorption in the UV range, despite its low carrier mobility [96]. Moreover, the photodetector based on (n-propylammonium)_2_CsAgBiBr_7_ single crystals exhibits a large on/off ratio (≈10^4^) and a high detection rate (5.3 × 10^11^ Jones) at 405 nm light [97]. Their study provides a potential avenue for the design of various perovskite-based photodetectors.

(2) The device performance needs to be further improved. The ideal photodetector should have excellent spectral response range tunability, high responsivity, high sensitivity, low noise, and high stability. Looking into the future, research on how to improve the performance of lead-free double perovskite photodetectors can start from four aspects, including reducing dark current, increasing response speed, fabricating the wide-spectrum photodetector, and engineering the interface of the devices.

First, a lower dark current is a prerequisite for high detectivity. For semiconductor-based photodetectors, the dark current is closely related to the defect density of the materials. However, the crystal defects of lead-free double perovskite are much more numerous than those of lead-based perovskite. For example, the Bi_Ag_ and halogen vacancies in Bi^3+^-based double perovskite and In_Ag_ in In^3+^-based double perovskite are deep electron traps, and these unnecessary deep-level defects strongly affect the carrier density and transport. In the meanwhile, the direct contact between the perovskite layer and the functional layer can cause interface recombination loss, and the combined effects of these factors will lead to a higher dark current in the device. Optimizing the growth conditions or metal ion doping can suppress internal defects in the crystal, while introducing a passivation layer on the surface of perovskite can eliminate the surface defects. These strategies can effectively reduce the dark current of the device and improve the detectivity. For example, doping Rb^+^ in the Cs_2_AgBiBr_6_ crystal can improve its response to X-rays due to the extended carrier lifetime, significant reduction of dark current, and polarization formation [98].

Second, in optical communication and time-of-flight imaging applications, the high response speed of detectors is essential. In theory, reducing the time required for carrier diffusion and charge collection helps to obtain high-speed photodetectors. When designing lead-free double perovskite-based photodetectors, the strategy of shortening the distance between electrodes can improve charge collection efficiency. For detectors with vertical structures, reducing the thickness of the semiconductor layer can achieve a rapid response of the device.

Third, the research of wide-spectrum photodetectors based on lead free double perovskite will be an important research field for future photodetectors. The narrow bandgap characteristic of perovskite is the primary condition for achieving wide spectral detection. For Sb^3+^-based, Ti^4+^-based, Fe^3+^-based, Au^3+^-based, and Sn^4+^-based double perovskite, their bandgaps are relatively small, making them suitable candidates for manufacturing wide spectral detectors. For Bi^3+^-based, In^3+^-based, and other large-bandgap double perovskite, energy band engineering is an effective way to adjust their bandgaps and design suitable carrier leap modes. Another commonly used strategy for achieving broadband detection (in the visible and near-infrared regions) is to integrate perovskite with low-bandgap polymers or organic small molecules, such as CyPF_6_, Cy1BF_4_ [99], NDI-DPP [100], PTB7-Th [101], and so on. For example, a broadband detector can be fabricated by combining perovskite with low-bandgap PDPPTDTPT, which shows a spectral response range of 350 nm to 1050 nm and an ultrafast response rate of 5 ns at a wavelength of 800 nm [102].

Finally, to achieve high-performance photodetectors, interface engineering is needed to improve the charge separation and extraction rates [103]. In optoelectronic devices, solution-based prepared perovskite films have many pinholes and surface defects, and the perovskite materials are usually sensitive to the surface conditions of adjacent layers, which requires the introduction of appropriate insertion layers to alleviate the impact of these unfavorable factors on device performance [104]. Introducing functionalized interlayers can promote favorable interface charge dynamics and minimize the carrier loss of interfacial dipoles, ultimately improving device performance. For example, F4-TCNQ layer (2,3,5,6-tetrafluoro-7,7,8,8-tetracyanoquinodimethane) was introduced between NiO_x_/perovskite layers, and it can increase the hole concentration and work function of NiO_x_ HTL and thus can improve hole extraction and carrier mobility [105]. Functionalized interlayers, such as the ALD-MO_x_ interlayer mentioned before, can also significantly optimize the quality of perovskite films. In addition, a suitable interface layer facilitates the ideal energy band alignment between the perovskite and the transport layers. Currently, the ETLs used for perovskite photodetectors are usually metal oxides such as ZnO, TiO_2_, and SnO_2_. However, at the ETL and perovskite interface, we usually observe non-radiative recombination and low electron extraction efficiency, so it is necessary to introduce an additional interface layer to alleviate these problems. For example, ZrCl_4_ can modify the TiO_2_ ETL by eliminating the offset between the conduction band edge of the TiO_2_ transport layer and the absorber and improving the charge extraction efficiency; thus, perovskite solar cells based on modified ETL achieved a much higher stable efficiency [106]. Regarding interface engineering, future research can focus on developing multifunctional molecular interface materials that can simultaneously passivate the defects, enhance the device carrier extraction, and extend the device lifetime [107]. We believe that the performance of lead-free double perovskite-based photodetectors will improve gradually and move closer to commercialization as more and more researchers are involved.

Based on the above discussions, although the performance of lead-free double perovskite photodetectors may be inferior to that of lead halide devices, there is no doubt that lead-free double perovskite has unlimited potential for applications in optoelectronic devices such as photodetectors. With a deeper understanding of the fundamental physical and optical properties of lead-free double perovskite, we can completely achieve photodetectors with stable performance, environmental friendliness, and high commercial value. The technology of lead-free perovskite photodetectors is young and promising, and we believe that its developmental path will become more and more open and bright.

## Figures and Tables

**Table 1:** Summary of key parameters of photodetectors based on lead-free double perovskite.

Device Configuration	Spectral Range (nm)	R (A W^−1^)	D* (Jones)	Response Time (ms)	On/Off Ratio	Ref.
Au/Cs_2_AgBiBr_6_/Au	300–800	7.01	5.66 × 10^11^	0.956/0.955	2.2 × 10^4^	[12]
ALD-NiOx modified FTO/Cs_2_AgBiBr_6_/TiO_2_/Au	350–550	-	1.2 × 10^13^	-	-	[13]
FTO/TiO_2_/Cs_2_AgBiBr_6_/CuSCN/Au	300–600	0.34	1.03 × 10^13^	28.75/32.95	-	[17]
FTO/SnO_2_/ZnO/Cs_2_AgBiBr_6_/Au	-	0.608	2.97 × 10^10^	124/61	-	[18]
FTO/Cs_2_AgBiBr_6_/Au	350–500	9.8	-	1.2 × 10^−3^/0.5 × 10^−3^	-	[25]
Au/Cs_2_AgInCl_6_/Au	340–400	0.97	~10^12^	0.8/1.0	~500	[50]
FTO/Cs_2_SnI_6_/FTO	-	0.006	2 × 10^9^	-	-	[61]
FTO/c-TiO_2_/Cs_2_SnI_6_/Spiro OMeTAD/Au	300–1000	0.001	6.03 × 10^10^	590/190	151	[64]
FTO/TiO_2_/Cs_2_SnI_6_-Ni^3+^/TiO_2_/FTO	350–950	160	4.52 × 10^12^	-	-	[65]
FTO/TiO_2_/Cs_2_SnI_6_-Zn^2+^/TiO_2_/FTO	350–900	710	1.56 × 10^13^	190/530	-	[65]
ITO/Rb_2_TeI_6_/ITO	450	0.0014	10^10^	16.4/19.2	-	[72]
Au/(4FPEA)_4_AgBiI_8_/Au	400	0.002	5 × 10^8^	-	-	[76]
Au/(4FPEA)_4_AgBiI_8_/Au	400	0.01	6 × 10^9^	-	-	[76]
ITO/Cs_2_AgBiBr_6_/SnO_2_/Au	350	0.11	2.1 × 10^10^	2	-	[77]
SnO_2_/Cs_2_AgBiBr_6_/TFB/Au	300–550	0.14	3.3 × 10^12^	1.7 × 10^−5^	-	[78]
FTO/Cs_2_SnI_6_/FTO	500–900	-	-	100/100	-	[79]
MWCNT/Cs_2_SnCl_6_:Bi/GaN	350–400	0.208	1.2 × 10^12^	7.5 × 10^−4^/9.1 × 10^−4^	-	[80]
In/GaN/Cs_2_AgBiBr_6_/Ag	200–550	1.46	9.4 × 10^12^	3.463/8.442	-	[81]
Au/Cs_2_AgBiBr_6_ microplatelets/Au	450	0.245	1.3 × 10^11^	145 × 10^−3^/136 × 10^−3^	2.8 × 10^3^	[82]
Au/MA_2_AgBiBr_6_ microplatelets/Au	450	0.058	2.9 × 10^10^	-	281	[82]
ITO/Cs_2_PdBr_6_/Ag	-	-	-	-	-	[83]
Flexible ITO/SnO_2_/Cs_2_AgBiBr_6_/Carbon	-	0.031	8.04 × 10^11^	-	0.5 × 10^4^	[84]
ITO/Cs_2_AgBiBr_6_/Ag	375	-	-	6.13 × 10^−3^/28.02 × 10^−3^	6.6 × 10^3^	[85]

## Data Availability

Not applicable.
